# Novel Pseudo-Wavelet Function for MMG Signal Extraction during Dynamic Fatiguing Contractions

**DOI:** 10.3390/s140609489

**Published:** 2014-05-28

**Authors:** Mohamed R. Al-Mulla, Francisco Sepulveda

**Affiliations:** 1 College of Computing Science and Engineering, Kuwait University, P.O. Box 5969, Safat 13060, Kuwait; 2 School of Computer Science and Electronic Engineering, University of Essex, Colchester CO4 3SQ, UK; E-Mail: fsepulv@essex.ac.uk

**Keywords:** genetic algorithms, localised muscle fatigue, mechanomyography, wavelet analysis, pseudo-wavelets

## Abstract

The purpose of this study was to develop an algorithm to classify muscle fatigue content in sports related scenarios. Mechanomyography (MMG) signals of the biceps muscle were recorded from thirteen subjects performing dynamic contractions until fatigue. For training and testing purposes, the signals were labeled in two classes (Non-Fatigue and Fatigue). A genetic algorithm was used to evolve a pseudo-wavelet function for optimizing the detection of muscle fatigue. Tuning of the generalized evolved pseudo-wavelet function was based on the decomposition of 70% of the conducted MMG trials. After completing 25 independent pseudo-wavelet evolution runs, the best run was selected and then tested on the remaining 30% of the data to measure the classification performance. Results show that the evolved pseudo-wavelet improved the classification rate of muscle fatigue by 4.70 percentage points to 16.61 percentage points when compared to other standard wavelet functions, giving an average correct classification of 80.63%, with statistical significance (*p* < 0.05).

## 1. Introduction

Studies on localized muscle fatigue have focused mainly on the decline in the force of a muscle contraction during a sustained activity [1], which results in a definition of fatigue as the inability of a muscle to continue exerting force or power. There are various techniques to detect muscle fatigue, although the most researched ones are mechanomyography (MMG) and surface electromyography (sEMG) [2]. In MMG it is the mechanical signal from the surface of a contracting muscle that is measured, *i.e.*, when the muscle fibers move they cause vibrations which can be recorded [3]. A selection of sensors can be utilized to record MMG signals, e.g., hydrophones, condenser microphones, piezoelectric contact sensors, accelerometers, and laser distance sensors [3,4]. An accelerometer is utilized to record both dynamic and static forces. In studies on muscle fatigue accelerometers have been utilized to detect changes during exercise. Barry *et al.* [5] used an accelerometer to detect changes in vibration amplitude during voluntary and evoked muscle vibrations in fatiguing muscle contractions. Evoked muscle vibrations were triggered by supramaximal percutaneous ulnar nerve stimulation. Their findings suggested a relationship in the fatiguing muscles between the vibration amplitude from evoked muscle twitches and the evoked twitch force. A correlation was found between the vibration from evoked muscle twitches and vibration measurements during voluntarily contractions.

MMG signal detection can be applied to muscle activity in both dynamic (*i.e.*, non-isometric) and isometric contractions. Research on localized muscle fatigue in dynamic contractions has investigated MMG amplitude in both concentric [6,7] and eccentric muscle contractions [8]. Several studies on rectus femoris and vastus lateralis muscles (working at maximal concentric isokinetic leg extension at different velocities) have identified a linear relationship between the MMG amplitude and the work load. Researchers also found that in the vastus medialis muscle, there was a quadratic decrease in MMG amplitude in relation to the work load [6]. The decrease in MMG amplitude can been explained by the so-called “muscle wisdom”, *i.e.*, where the fatiguing muscle is activated more economically as the central nervous system reduces the motor unit firing rate to compensate for muscle fatigue, which in turn reduces the number of pressure waves recorded by MMG [9]. An alternative explanation is that muscle elasticity is reduced due to intramuscular pressure, muscle thickness and fluid content increasing over time in both static and dynamic contractions, which influences the muscle oscillations and pressures waves recorded by the MMG [10–12]. However, in another study, Sorgaard [13] argued that intramuscular pressure does not have an affect on the MMG amplitude. After studying muscle actions from the biceps brachii at various maximum voluntary contraction (MVC) levels during isometric ramp contractions, they found that even when the intramuscular pressure was increased, the MMG amplitude showed a linear relationship with force. Nevertheless, more research is needed for various muscle groups and different torque levels to provide sufficient evidence to support this suggestion.

By using a wavelet function (WF), the wavelet transform (WT) decomposes a signal into numerous multi-resolution components [14,15]. It is used to detect and characterize the short time components within a non-stationary signal, providing information regarding the signal's time-frequency. The MMG signal is high-dimensionally chaotic, and should therefor by analyzed using non-linear dynamics, such as wavelet functions [16]. Several studies have used wavelet functions for analyzing the MMG signal in localized muscle fatigue. Beck *et al.* [17] have used wavelet analysis on the MMG signal utilizing the frequency space by multiplying each of the wavelets with the Fourier transform of the signal, where they converted both the real and imaginary wavelet transformed signals into the time domain with the inverse Fourier transform. Findings of this research demonstrated muscle-specific differences for each of the wavelet bands with various percentages of MVC in isometric contractions for the total MMG intensity values.

To process MMG signals emanating from dynamic muscle contractions, various research has looked at wavelet-based methods to deal with the stochastic nature of the MMG signal. Beck *et al.* [18] have proposed a new wavelet based technique for MMG signal processing where the wavelet analysis utilities a filter bank containing 11 non-linearly scaled wavelets, in which the optimal relationship between time and frequency resolutions is maintained. These wavelets were scaled in a nonl-inear fashion, allowing them to give equal weight to low and high frequencies of the MMG signals. The new method provides information about the MMG intensity patterns, which can be used for statistical pattern recognition of MMG signals [19]. According to Beck *et al.*, the most suitable method for signal processing of MMG signals from dynamic muscle contractions is their developed wavelet-based methods since they do not expect signal stationary [17]. Ryan *et al.* [20] conducted a study comparing patterns of responses from the MMG center frequency analyzed with short-time Fourier transform (STFT) and continuous wavelet transform (CWT). Their findings showed similarities in pattern responses between CWT and STFT. This falls in line with previous research by Beck *et al.* [17], as mentioned above. Armstrong [21] utilized the intensity analysis developed by von Tscharner [22] to investigate the effects of fatigue and to evaluate postural control during single-legged stance. The intensity analysis describes the power of a stochastic signal as a function of both time and frequency, however, Armstrong amended this analysis method by applying a filter bank of 11 Morlet wavelets. His results indicate that this intensity analysis is a useful method in studying fatigue and postural control using MMG signals.

In another study, Tarata [23] investigated the most suitable wavelet transform to provides all the information within the MMG signal from static (*i.e.*, isometric) contractions. Signals from dynamic contractions can only be characterised with parameters that are computed on very short time scales, which would display the transient parts of the contracting muscle. In their research CWT was selected instead of DWT as it preserves all the information in the signal. The “Mexican Hat” wavelet was the most suited out of a set of wavelets, based on its higher sensitivity, which was computed on the ratio of variation of IMS and IMedS, over their maximal value.

There has been little research on classification of the fatigue content in MMG signals. Most research on classification of MMG signals in muscle activity are used for prosthetic control. Xie *et al.* [24] proposed a method using the classification results of Short-Time Fourier Transform (STFT), Stationary Wavelet Transform (SWT), and S-Transform (ST) combined with Singular value decomposition (SVD), to find the highest classification accuracy of hand movements based on MMG signals, which gave a classification performance of 89.7% between the two classes (wrist flexion and wrist extension). Another research has also classified the MMG signal emanating from muscle activity for prosthetic control, getting classification accuracy of 70% between the two classes (flexion and extension). However, that research [25] did not use wavelet transform for classification purposes, but rather used RMS-based (Root Mean Square) as a feature for classification.

Various research has used different classification techniques for sEMG signals in localized muscle fatigue, which may also be applicable to research on MMG signals. These include genetic programming and genetic algorithms [26–29], statistical analysis [30–32], as well as classification methods to predict fatigue by using neural networks [33] or linear discriminant analysis (LDA) [34]. A variation of these techniques have been adapted in this research to evolve a pseudo-wavelet for classifying fatigue content in the MMG signal. MMG was selected as the signal acquisition method in this study as MMG has less hindrances and errors in the signal acquisition and signal processing than sEMG [35]. In addition, MMG requires fewer hardware with more reliable measuring devices which increase accuracy in the signal detection system.

## 2. Methods

This research utilized wavelet analysis to take into account the stochastic and transitory nature of the MMG signal. In addition, a genetic algorithm was chosen as the method to provide an optimal solution by tuning a pseudo-wavelet function for its optimal decomposition of MMG targeted in extracting muscle fatigue content. In the end, the evolved pseudo-wavelet was validated and compared with other common wavelet transforms. The term “pseudo-wavelet” is used here to indicate that the evolved wavelet-like function is not required to meet the necessary conditions (e.g., admissibility and regularity) to be formally described as a wavelet. Pseudo-wavelets are thus a convenient joint time-frequency tool aimed specifically at pattern recognition.

### 2.1. Data Recording and Pre-Processing

Thirteen athletic, healthy male subjects (mean age 27.5 ± 3.6 years) volunteered for this research. The study was approved by the University of Essex's Ethical Committee and all subjects signed an informed consent form prior to taking part in the study.

The participants, all non-smokers, were seated on a “preacher” biceps curl machine to ensure stability and biceps isolation while performing biceps curl tasks. The participants reached physiological fatigue and was encouraged during the trial to reach the complete fatigue stage (unable to continue the exercise).

To evaluate the Maximum Dynamic Strength (MDS) percentage for each participant we used the average of three 100% MDS measurements on three different days to ensure correct estimation. The 100% MDS measurements for each subject were determined by the one-repetition maximum (1 RM), where the subjects managed to keep the correct technique while executing the repetition with the heaviest possible load on a preacher biceps curl machine. In other words 100% MDS is equal to 1 RM. Determining each subject's 100% MDS allowed estimating the correct loading MDS (40% MDS and 70% MDS) across subjects when conducting the trials.

After establishing the MDS for each subject the trials where carried out. After the warm-up period, all the thirteen participants carried out 3 trials of non-isometric exercises with 40% Maximum Dynamic Strength (MDS) and 3 trials of 70% MDS with a one week resting period between trials to ensure full recovery from the biceps fatigue, giving a total of 104 trials. Only one trial was performed per day for each subject in order to avoid injury.

The MMG signal was recorded using a 3-axis accelerometer (Biometrics, ACL300 (range ± 10 G)). The accelerometer was placed on the muscle belly of the biceps Brachii, without covering the end plate zone or getting too close to the musculotendinous region [36]. A flexible electrogoniometer (Biometrics Ltd., Newport, UK) was placed on the lateral side of the arm to measure the elbow angle and arm oscillations.

**Figure 1.**
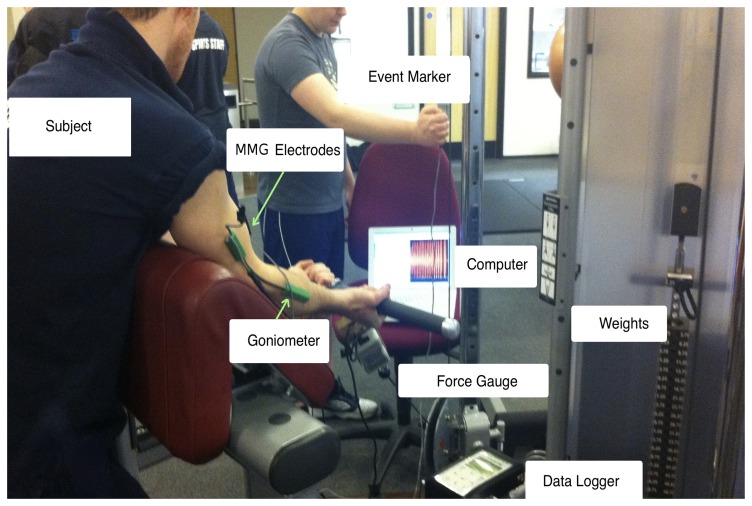
Experimental set-up showing one of the trials.

The test bed set up for one of the conducted trials is shown in Figure 1.

### 2.2. Labelling the Signals

The recorded MMG signals were grouped into Fatigue and Non-Fatigue epochs. Initial recordings in the first few repetitions when the subjects felt “fresh” were considered “Non-Fatigue”, and when the subject was unable to perform the sustained task the epochs were labeled as Fatigue, as per [37]. In the signal analysis, the first repetition was therefore labeled as Non-Fatigue, while the last repetition was labeled as Fatigue. This information was then used to train and test the classifier.

### 2.3. Wavelet Decomposition

Wavelet transform has a variation of standard mother wavelet functions that are used to decompose the signal. Some of these mother wavelet are Morlet, Symmlet, Mexican Hat, Daubechies *etc.* [37]. Although there are no specific rule for which wavelet is most suited for a signal, there are certain guidelines for the selection of a wavelet, e.g., Db4 is said to be suited for signals using feature extractions and linear approximation with more than four samples, while Db6 is used for a signal approximated by a quadratic function over the support of six; coiflet6 is better suited for data compression results [38].

In general, however, in order to determine the most suited wavelet, the properties of the wavelet function and the characteristic of the signal need to be analyzed and matched for specific data sets.

Evolved pseudo-wavelets are simply a function-fit approach to wavelet lifting that is based on genetic algorithms. The idea is to obtain an joint time-frequency transformation that is optimized for class separation rather than the usual optimization aimed at perfect reconstruction. However, in the evolutionary process the original wavelet may no longer meet the mathematical requirements to be called a “wavelet” proper (in particular, the zero integral and vanishing moments requirements cannot be guaranteed), hence the “pseudo-wavelet” term. From a signals and systems point of view, this also means that the evolving the transfer function (*i.e.*, the wavelet) applied to an input signal no longer guarantees conservation of energy, but this is not an issue for pure pattern recognition purposes (it would be an issue in signal modeling and transformation reversibility scenarios). Nonetheless, the pseudo-wavelet approach still preserves the multi-resolution properties of standard wavelets, which is the main reason for trying this approach.

The pseudo-wavelet developed in this research uses scaling function (phi) coefficients that are best suited to find the optimal shape for our application. The aim was to develop a custom-made wavelet-like shape suitable for join-time frequency decomposition for muscle fatigue detection in the MMG signal. Random values for the scaling function coefficients were first used that were evolved by the GA. Ten coefficients for phi was then selected.

### 2.4. Genetic Algorithms

Genetic Algorithms (GA) are useful tools to solve linear and nonlinear problems, using operators such as mutation, crossover and selection operations applied to each individual in the population to explore the optimal solution is the state space [39]. Selecting GA to evolve a pseudo-wavelet or to modify a standard wavelet will presumably produce an optimal solution that discovers the shape of a (pseudo)wavelet for better, data-specific joint-time frequency decomposition that detects muscle fatigue within the MMG signal. Out of the 104 trials, 70% were used for the testing phase, while the remaining 30% were utilized in the testing phase.

**Figure 2.**
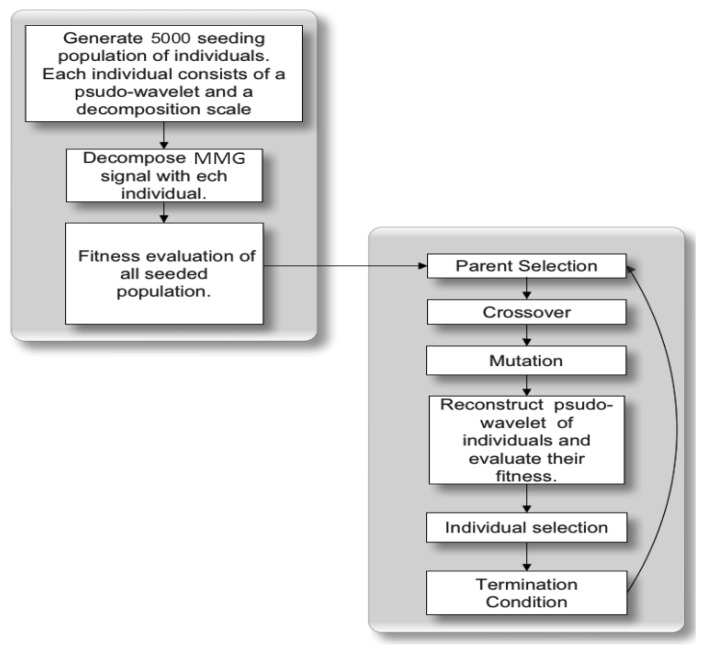
Flowchart of the pseudo-wavelet evolution.

Figure 2 shows a flow chart with the steps taken for the initialization and running of the GA. Table 1 displays the parameter setting for the GA runs.

#### 2.4.1. Solution Representation

The solution representation was used to find the optimal wavelet by utilising standard wavelet functions, such as Mexican hat, Daubechies, Symlet, *etc*. The evolved pseudo-wavelet uses scaling function (phi) coefficients from 1 to 19. Normally a scaling function of 1–10 is chosen, although previous studies by Kumar *et al.* argue that muscle fatigue content lays between scale 9 and 10 [37]. The GA in this research was given a wide range (1–19) to determine the most optimal scale for class discrimination.

#### 2.4.2. Fitness Function

A fitness function in the GA is utilized to discover the optimal solution in the search space. In this research the modified Davies Bouldin Index (DBI) was chosen in the fitness function, due to the DBIs simplicity and effectiveness. Data cluster linear overlap was calculated using the modified DBI [40] by determining the ratio of intracluster spread to intercluster centroid distance. Smaller DBI values indicate better class separation.

The joint-time frequency decomposition by the pseudo-wavelet was obtained for every scale (1–19) and extracted in one second intervals to determine the DBI between the two classes (*i.e.*, Fatigue and Non-Fatigue). This again helped the evolutionary processes by intending to minimize the DBI, which then allows the fitness function to increase the separation between the two classes. Usually the fitness function operates by maximization, utilizing a hill climbing technique. This was enabled by the DBI being transformed into negative numbers, allowing the fitness function to use the hill climbing method by attempting to bring the (now) negative DBI closer to zero.

## 3. Validation/Classification

For a comparison between the evolved pseudo-wavelet and other commonly used wavelet functions, LDA (linear discriminant analysis) was chosen due to its simplicity, being well established and light on computational resources. The decomposed MMG signal from the pseudo-wavelet was the input for the training and testing phase of the LDA classifier. As was the case in the evolutionary process, the classifier was trained using 70% the trials, followed by testing with the remaining 30% of the trials.

It must be noted that the decomposition scale value of the eight compared standard wavelet functions (see Wavelet Decomposition above) matched the decomposition scale value of the evolved pseudo-wavelet function, enabling a meaningful comparison.

## 4. Results

This research has several interesting results. Firstly, the GA selected the optimal wavelet for MMG classification, as well as the optimal scale for decomposing the MMG signal. Also, the classification performance of the evolved pseudo-wavelet proved to be better than traditional wavelet functions for MMG classification.

**Figure 3.**
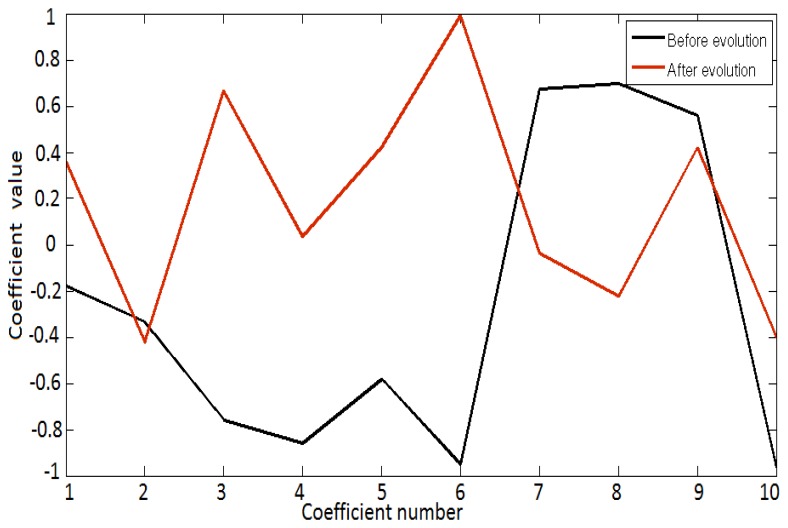
Pseudo-wavelet before and after evolution.

The optimal wavelet was selected by the GA based on the solution representation, where it finds the improvements according to the fitness function of the final evolved population with the best DBI scoring, which can be seen in Figure 3. Figure 3 shows superimposed shapes of original randomly generated pseudo-wavelets with the final pseudo-wavelet at the end of a typical evolutionary process.

Another observation in this research was the correlation between the shape of the wavelet and the optimal scale. The shape of the wavelet has an effect on the selection of the optimal scale to best discriminate between Fatigue and Non-fatigue content of the MMG signal. This finding falls in line with Kumar *et al.* [37] result that certain wavelet functions at certain scales can best contrast between Fatigue and Non-Fatigue, although in their case this was based on sEMG signals.

**Figure 4.**
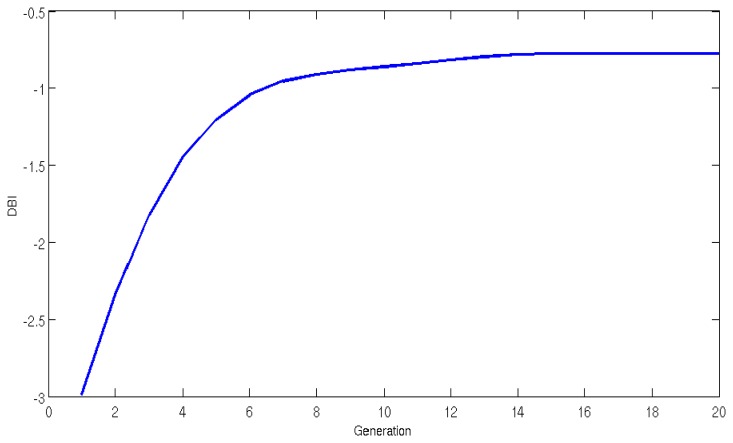
Generation fitness during the GA.

In the present study, it was the GA that selected the optimal scale based on the wavelet function rather than a human selecting the most recommended wavelet functions for fatigue content analysis. By utilizing the DBI, the GA selected the most suitable scale for decomposing the MMG signals. The optimal scale finds the best separability between the fatigue classes (Fatigue and Non-Fatigue). Figure 4 shows the improvements in the pseudo-wavelet population fitness (values closer to zero indicate improved fitness) accomplished by one of the GA runs in optimizing the pseudo-wavelet function and the most optimal scale.

The GA was initialized with 5000 individuals with randomly generated coefficients. During the first generation the GA run was seeded with relatively good solutions averaging a transformed DBI of −2.99. After proceeding with the evolutionary process the fitness improved for this particular case and reached its optimal range of −0.775 DBI, around the 16th generation.

The GA initialization and GA run were repeated 25 times using different epochs every time to ensure optimal coverage of the GA search space. Table 2 shows the 25 independent GA runs. From this table, consistency in the results from each GA run can be initially observed.

It can be seen that the optimal scale is 9, which gives a separability of −0.775. This gives an indication that the GA is able to separate the MMG signals from the two different classes (Fatigue and Non-Fatigue).

In the classification of the MMG signals, both the optimal wavelet and the optimal scale were utilised. The classification performance with the developed pseudo-wavelet was 80.63%. Compared to traditional wavelet functions, the pseudo-wavelet was able to better classify the MMG signal, getting an average of 80.63% (*p* < 0.05, Wilcoxon's rank sum test) *vs*. 75.94% for DB2, which was the second best wavelet function.

**Figure 5.**
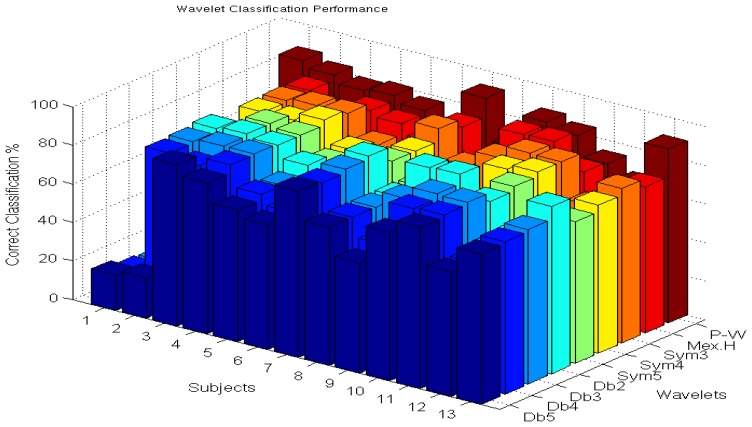
Graphical representation of the Classification performance (in %) (P-W = Pseudo-wavelet).

Table 3 shows a classification comparison of the evolved wavelet with 8 different wavelet functions in decomposing the MMG signal, hence enabling the benchmarking of the classification capabilities of the evolved pseudo-wavelet. Classification performance of all thirteen subjects with the unseen test data sets shows that the evolved pseudo-wavelet function has outperformed all of the other wavelets by a range between 4.70 percentage points and 16.61 percentage points, giving an average of 80.63%. Moreover the average for all the other wavelets combined gives 70.92% with significance of (*p* < 0.05) . When looking at the standard deviation across the classification averages, the evolved wavelet also showed the lowest values, which could be explained by its consistency in classification across subjects. All wavelet and pseudo-wavelet functions were used with scale 9 to ensure consistency in the comparisons. Figure 5 illustrates graphically the classification performance (in %) seen in Table 3.

## 5. Discussion

Developing new methods to classify the MMG signal is an interesting approach which is mainly used in the field of prosthetic control and muscle activity research. Using wavelet functions for MMG signal processing has been used by several researchers, in particular Beck *et al.* [41] who created a wavelet-based technique for pattern recognition of the MMG signal in muscle activity. Some researchers have found [7,42,43] that both Discrete and Continuous Wavelet transforms are most appropriate for analyzing signals of stochastic nature. The pseudo-wavelet developed in our research to classify the MMG signal were based on continuous wavelet transforms.

Various researchers have looked at using wavelet based analysis for MMG signal processing [7,18,41–44]. Beck *et al.* [17] developed a technique containing 11 nonlinear scaled wavelets to determine the optimal relationship between time and frequency resolutions, which can be used in statistical pattern recognition. In the present paper, another approach was developed where the GA selects the most appropriate pseudo-wavelet as well as the optimal scale for classifying the MMG signal. The method proved useful and demonstrated the pseudo-wavelet's ability to differentiate between Fatigue and Non-Fatigue content of the MMG signal.

The optimal scale for the evolved wavelet function in this research was 9, which was determined by the GA. This finding falls in line with research on sEMG where the optimal scale was 8 and 9 (out of 10 levels) to determine fatigue content using Sym4 or Sym5 [37].

From the comparison of the classification performance with other traditional wavelets, it shows that the evolved pseudo-wavelet significantly outperforms the other wavelets. According to Tarata [23], Mexican Hat is the most suited wavelet for MMG analysis. However, results shown here indicate that the evolved pseudo-wavelet gives even better results. This finding is worth noting for future research and the selection of which wavelet based method to use in MMG signal analysis.

Compared to previous research using a pseudo-wavelet to classify sEMG signal [29], the pseudo-wavelet utilised in this research produced similar results. This show that the methodology for developing the pseudo-wavelet is consistent and will produce similarly improved results in future research. The evolved pseudo-wavelet approach has proved to be an efficient method of classifying fatigue content in MMG signal, as had been the case in our previous study with sEMG.

## Figures and Tables

**Table 1.** Parameter settings for the GA runs.

**Parameter**	**Value**
Independent runs	25
Population size	5000
Maximum number of generations	20
Mutation probability	10%
Crossover probability	90%
Selection type	Tournament, size 5
Termination criterion	Maximum number of generations

**Table 2.** Twenty five independent runs, showing the best individual. Coef = Coefficient.

**Best Indiv.**	**Coef 1**	**Coef 2**	**Coef 3**	**Coef 4**	**Coef 5**	**Coef 6**	**Coef 7**	**Coef 8**	**Coef 9**	**Coef 10**	**Scale**	**DBI**
1	0.723961	−0.648377	0.506117	−0.997074	0.957322	−0.663827	0.474965	0.145197	−0.866139	−0.704656	11	−0.845966
2	0.171349	−0.630587	0.191098	−0.796508	−0.223069	−0.231696	−0.201590	−0.784462	−0.094844	0.754290	12	−0.849446
3	−0.621409	0.863039	0.092617	0.511425	0.834059	0.492856	0.892356	−0.328452	−0.866739	0.386239	19	−0.843199
4	−0.968087	0.513111	−0.698193	−0.638030	−0.415072	−0.120873	−0.006556	−0.076733	0.126581	−0.865160	13	−0.855289
5	−0.072398	0.085546	−0.067654	0.133409	0.705926	0.733416	0.960984	−0.893476	0.425220	0.350938	18	−0.887643
6	0.295927	−0.040205	−0.082915	−0.729146	0.486375	0.961391	0.954147	0.949864	0.911659	−0.985766	17	−0.883891
7	0.854157	0.218216	−0.256734	−0.862797	−0.274309	−0.934391	−0.976033	−0.121563	0.891731	−0.203497	18	−0.853443
8	0.470668	−0.698475	−0.075017	−0.021013	−0.153569	−0.976294	−0.759343	−0.776215	0.834345	0.323383	16	−0.860869
9	−0.166401	0.103557	0.328560	−0.377781	0.066326	0.728509	0.903813	−0.315157	0.304919	0.218099	19	−0.860301
10	−0.950290	0.075625	0.761739	0.947685	−0.515141	0.946812	−0.286644	0.268228	0.718876	0.578017	17	−0.807826
11	0.182845	0.800795	0.023454	0.876363	−0.950901	−0.524622	0.765737	0.122379	0.864829	−0.551023	18	−0.822942
12	−0.041094	0.578249	−0.574139	−0.740829	0.251840	−0.868452	−0.306431	−0.957537	0.951027	0.219532	19	−0.803427
13	0.358269	−0.425574	0.665891	0.034365	0.420418	0.991693	−0.038982	−0.224130	0.419404	−0.400792	9	−0.775413
14	0.788058	−0.110733	0.770510	0.875107	−0.441378	0.558319	0.959235	−0.842636	0.652069	0.946038	18	−0.800586
15	−0.063364	−0.694161	−0.851712	−0.013747	0.104839	−0.113733	−0.305231	0.089397	0.828311	−0.122823	17	−0.789409
16	0.986021	−0.084163	−0.231627	−0.302683	−0.643388	−0.749955	−0.641338	−0.804129	0.766124	−0.627568	19	−0.869848
17	−0.782537	0.058527	0.328714	−0.377569	0.967579	0.569102	0.967395	0.387475	0.872844	−0.537900	19	−0.839118
18	−0.192894	0.379860	−0.800941	0.059314	0.964982	0.482363	0.994087	0.950543	0.369976	0.281219	19	−0.860854
19	−0.259580	0.192873	−0.140098	0.349956	−0.431673	0.889820	0.243234	0.525703	0.737410	−0.777512	19	−0.850126
20	−0.617202	0.316896	0.159165	0.476439	0.279625	0.731769	0.764929	−0.596646	−0.967892	0.907438	18	−0.783482
21	−0.720375	−0.976723	0.297287	−0.930802	0.938883	−0.940783	−0.570633	−0.404012	−0.238041	−0.428351	10	−0.808680
22	0.227532	−0.636046	−0.282654	0.892203	−0.176587	0.929766	0.566680	0.825936	−0.345618	0.334357	17	−0.885709
23	−0.429893	−0.342897	0.979843	−0.335449	0.6333817	0.738707	0.2581927	0.512073	0.114374	0.751500	16	−0.886985
24	0.399314	0.133859	−0.845725	0.557674	−0.251278	−0.899172	−0.682572	0.078188	0.518772	0.120541	18	−0.875824
25	−0.572762	−0.484504	0.128970	0.964342	0.861597	0.987382	0.480856	−0.301787	0.306427	0.342617	19	−0.866100

**Average**	−0.040007	−0.058092	0.013062	−0.017806	0.159872	0.148724	0.205679	−0.102878	0.329425	0.012366	17	−0.842655

**Std.**	0.574208	0.498946	0.514651	0.655474	0.588575	0.757654	0.664933	0.584961	0.591062	0.584045	3	0.034297

**Table 3.** Classification Results (P-W = Pseudo-wavelet).

**Subjects**	**Db5 %**	**Db4 %**	**Db3 %**	**Db2 %**	**Sym5 %**	**Sym4 %**	**Sym3 %**	**Mexican Hat %**	**P-W%**
Subject 1	18.391	15.517	14.943	14.943	17.241	19.540	14.943	59.195	87.356
Subject 2	20.979	80.420	81.119	82.517	18.881	80.420	81.119	81.119	83.916
Subject 3	83.908	78.161	82.759	83.908	83.908	80.460	82.759	58.621	79.885
Subject 4	78.358	82.463	82.836	82.090	82.090	84.701	82.836	78.731	81.343
Subject 5	68.313	71.193	70.370	75.720	72.016	72.840	70.370	76.132	77.778
Subject 6	64.773	65.909	67.045	69.318	64.773	64.773	67.045	70.455	70.455
Subject 7	86.235	85.425	87.045	88.664	80.567	80.567	87.045	82.591	92.308
Subject 8	71.574	71.574	71.574	71.574	71.574	71.574	71.574	52.284	70.558
Subject 9	57.333	61.333	80.000	90.667	50.667	58.667	80.000	86.667	88.000
Subject 10	76.984	84.127	86.508	91.270	71.429	83.333	86.508	87.302	84.921
Subject 11	83.721	84.884	86.047	81.395	83.721	86.047	86.047	75.581	74.419
Subject 12	64.479	64.093	67.181	68.340	61.776	59.073	67.181	60.232	67.181
Subject 13	78.022	79.121	80.220	86.813	73.626	76.923	80.220	75.824	90.110

**Average**	65.621	71.094	73.665	75.940	64.021	70.686	73.665	72.672	80.633

**Std.**	22.115	18.610	19.044	19.891	22.476	17.923	19.044	11.551	8.107
